# Repetitive Sampling and Control Threshold Improve 16S rRNA Gene Sequencing Results From Produced Waters Associated With Hydraulically Fractured Shale

**DOI:** 10.3389/fmicb.2020.536978

**Published:** 2020-09-11

**Authors:** Jenna L. Shelton, Elliott P. Barnhart, Leslie Ruppert, Aaron M. Jubb, Madalyn S. Blondes, Christina A. DeVera

**Affiliations:** ^1^Eastern Energy Resources Science Center, U.S. Geological Survey, Sacramento, CA, United States; ^2^Wyoming-Montana Water Science Center, U.S. Geological Survey, Helena, MT, United States; ^3^Center for Biofilm Engineering, Montana State University, Bozeman, MT, United States; ^4^Eastern Energy Resources Science Center, U.S. Geological Survey, Reston, VA, United States

**Keywords:** low biomass samples, 16S/18S ribosomal RNA gene analysis, produced water, blanks, hydraulic fracturing

## Abstract

Sequencing microbial DNA from deep subsurface environments is complicated by a number of issues ranging from contamination to non-reproducible results. Many samples obtained from these environments – which are of great interest due to the potential to stimulate microbial methane generation – contain low biomass. Therefore, samples from these environments are difficult to study as sequencing results can be easily impacted by contamination. In this case, the low amount of sample biomass may be effectively swamped by the contaminating DNA and generate misleading results. Additionally, performing field work in these environments can be difficult, as researchers generally have limited access to and time on site. Therefore, optimizing a sampling plan to produce the best results while collecting the greatest number of samples over a short period of time is ideal. This study aimed to recommend an adequate sampling plan for field researchers obtaining microbial biomass for 16S rRNA gene sequencing, applicable specifically to low biomass oil and gas-producing environments. Forty-nine different samples were collected by filtering specific volumes of produced water from a hydraulically fractured well producing from the Niobrara Shale. Water was collected in two different sampling events 24 h apart. Four to five samples were collected from 11 specific volumes. These samples along with eight different blanks were submitted for analysis. DNA was extracted from each sample, and quantitative polymerase chain reaction (qPCR) and 16S rRNA Illumina MiSeq gene sequencing were performed to determine relative concentrations of biomass and microbial community composition, respectively. The qPCR results varied across sampled volumes, while no discernible trend correlated contamination to volume of water filtered. This suggests that collecting a larger volume of sample may not result in larger biomass concentrations or better representation of a sampled environment. Researchers could prioritize collecting many low volume samples over few high-volume samples. Our results suggest that there also may be variability in the concentration of microbial communities present in produced waters over short (i.e., hours) time scales, which warrants further investigation. Submission of multiple blanks is also vital to determining how contamination or low biomass effects may influence a sample set collected from an unknown environment.

## Introduction

Microbial generation of methane occurs in many terrestrial environments. Recent interest has focused on microbial communities in deep subsurface hydrocarbon reservoirs as they can be stimulated to produce additional natural gas from residual organic material in crude oil, coal, and/or shale reservoirs (Schlegel et al., 2013; Wuchter et al., 2013; Larter et al., 2015; Ritter et al., 2015; Daly et al., 2016; Barnhart et al., 2017). However, these environments typically contain low biomass concentrations due to inherent reservoir characteristics: low concentrations of essential nutrients, high temperatures, brackish to brine salinity conditions, high pressures, and low water drives (e.g., Head et al., 2003; Silva et al., 2013; Cai et al., 2015; Gieg, 2018). Unfortunately, field campaigns to collect samples can be complicated by associated expenses, access to wells from operators, and limited field access. Importantly, most researchers cannot determine parameters such as biomass concentrations prior to completing field sampling of hydrocarbon wells and may be left with samples that may be compromised or of low quality. Therefore, understanding the microbial constraints and controls on stimulating methanogenesis is challenging because identifying the microorganisms innate to these environments with field-based studies can be difficult with low biomass concentrations or other sampling issues, such as short time scale (e.g., days) microbial population changes (e.g., Zelaya et al., 2019) and the challenging and complex nature of produced water composition.

Low biomass concentrations have been identified in many environments outside of deep hydrocarbon reservoirs, such as those associated with subsurface sediments (Ogram et al., 1995), carbonate caves (Barton et al., 2006), spacecraft assembly cleanrooms (Vaishampayan et al., 2013), acidic, arsenic-rich creeks (Giloteaux et al., 2010), and subseafloor ocean crust (Santelli et al., 2010). However, studies on how the low-biomass characteristic impacts microbial sequencing are limited (e.g., Salter et al., 2014; Glassing et al., 2016). In these environments, many specific challenges with generating 16S rRNA gene data from sediment, rock, fluid or other materials have been identified. Irreproducible or low-quality DNA extraction is one common barrier to sequencing data from these environments, resulting in unconvincing results. Many researchers are developing tools or methods to deal with low-biomass results, such as modifying DNA extraction techniques (e.g., Webster et al., 2003; Barton et al., 2006), creating filters or other software that target contaminants via bioinformatics (e.g., Minich et al., 2018; Karstens et al., 2019), analyzing non-reproducible data (e.g., Chandler et al., 1997), and attempting to mitigate cross-contamination and contaminant DNA in samples (e.g., Eisenhofer et al., 2019). Laboratory contamination can occur via many routes, including contamination of extraction or PCR reagents and/or materials, surfaces, or human error (Salter et al., 2014; Glassing et al., 2016). Furthermore, variation in sequencing results have been observed across laboratories (e.g., Salter et al., 2014). Therefore, not only can samples from hydrocarbon wells possess low biomass, but they are also susceptible to contamination issues that are magnified by their innate low biomass nature. This means that biomass from contaminants may be proportional to sample biomass in low biomass samples but swamped by sample biomass in high biomass samples.

In this study, we collected biomass by filtering produced water from one hydraulically fractured well producing from the Niobrara Shale in northeastern Colorado. Hydraulic fracturing is a process where water, sand, and other chemicals are injected into a rock at a pressure great enough to fracture it, increase permeability, and stimulate hydrocarbon flow. The goal of the study was to ascertain a suitable sampling protocol for produced waters so that the highest quality data could be obtained in the most efficient way. We filtered specific volumes of water for biomass to determine how field measurements of 16S rRNA gene sequencing results vary across sample volume and if results from the samples from the same volume of filtered water were comparable. The hypothesis was that increasing volumes of water filtered would result in increasing concentrations of biomass collected. Our attempt was to simulate a situation where biomass concentrations are unknown and standard operating procedures are used to acquire data (e.g., non-low biomass specific DNA extraction methods) so that a researcher could use these results to determine the quality of the resulting 16S rRNA gene sequencing data. The specific research questions for this study were (i) do smaller volumes of sample result in sequentially smaller biomass concentrations; (ii) can field researchers use Cp (crossing point-PCR-cycle) values and blank samples to determine a quality threshold for low biomass samples; and (iii) can an ideal sampling plan be developed for researchers sampling low biomass produced waters. These results may help guide future sampling efforts in low-biomass environments to provide reproducible and quality data.

## Materials and Methods

### Field Methods

Produced water was collected in October 2018 from one hydraulically fractured oil and gas well producing formation water, oil, and gas from the Niobrara B Chalk in the Denver-Julesburg Basin. The well was located in Weld County, Colorado, United States. The operator and exact location of the well is confidential through a Technical Assistance Agreement with the operator. Water was collected from the well separator into six 5-L Nalgene HDPE carboys over a period of approximately 48 h, collecting a total of 30 L. As these carboys were unable to be autoclaved prior to field work, they were cleaned in the field according to USGS protocol by rinsing each carboy 3 times with sample water prior to filling the carboy to the brim (Graham et al., 2008). Carboy 1 was collected on day one, carboys 2 and 3 were collected concurrently on day two, and carboys 4, 5, and 6 were collected concurrently on day three.

When collecting the sample water, each triple rinsed carboy was filled to the brim (i.e., filled with no headspace), and closed tightly until filtration (to limit exposure to the atmosphere). First, we needed to determine the maximum amount of water that could be filtered before the filter clogged so that we could consistently filter a maximum volume of water without the filter clogging. Sterile Nalgene tubing was inserted into the mouth of a carboy and threaded through a peristaltic pump. A Sterivex GP Filter unit was attached to the other end of the tubing, and the pump was turned on. The filtrate (i.e., the water that passed through the filter) was measured using a graduated cylinder. The pump remained on until the filter clogged, and the volume of filtrate was then measured. The maximum volume of water that could be filtered was, on average, 1083 mL. Therefore, 1000 mL of filtrate was used as the maximum volume for this study.

**FIGURE 1:**
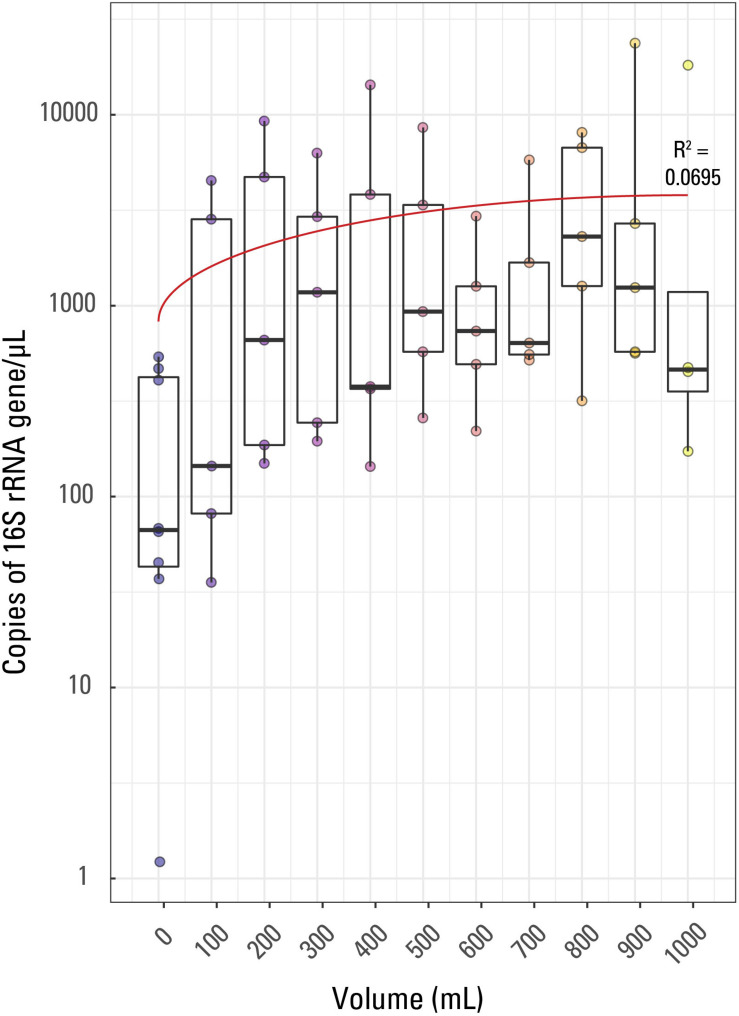
Boxplot of qPCR results. Volume of water filtered for each of the 57 samples is compared to the 16S rRNA copies/μL for each sample. The thick line in each box represents the median for each volume while the whiskers extend to roughly a 95% confidence interval. The red trendline has an *R*^2^ value of 0.0695. Samples are colored based on sample volume.

Fifty-seven filters were collected after filtering varied and specific volumes of filtrate. Volumes were selected that decreased sequentially from 1000 mL in an attempt to simulate changes in biomass concentrations. The following volumes of water were collected – 1000, 900, 800, 700, 600, 500, 400, 300, 200, 100, and 0 mL (Figure 1) – and at least four filters were collected at each given volume using the method described for determining the maximum volume of water described above. We attempted to remove any bias or error that may have been generated due to using 6 different carboys of sample water by randomizing the filters that came from each carboy. For example, all four of the 1000 mL filters were not generated by filtering water from the same carboy (see Table 1 for information about which samples came from each of the 6 carboys). This approach should eliminate any bias introduced by collecting water from different time points (i.e., potential differences in biomass concentrations across the carboys of water would be present across multiple volumes).

**TABLE 1:** Sequences per sample and OTUs identified per sample for each sample before and after contaminant removal.

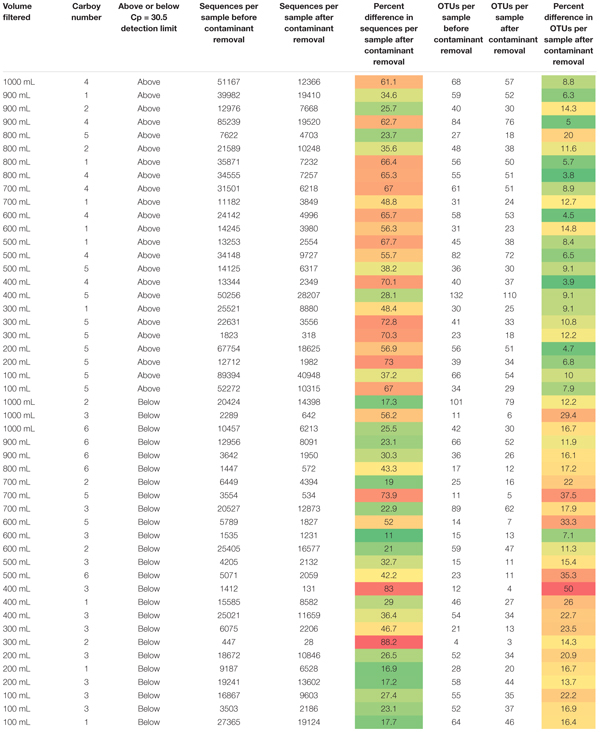

*Cells colored in red indicate a larger percent difference between the two measured values, while green-colored cells indicate a smaller percent change between the two measured cells.*

After a given volume of filtrate was reached, the filter was removed from the tubing, capped, and placed immediately on dry ice. A new sterile filter was then attached to the Nalgene tubing, and a different specific volume of water was filtered through that filter by repeating the above process. The Nalgene carboys were kept well-mixed during the filtration process by physically shaking the carboys. In addition to the samples discussed above, a total of eight different internal sample blanks were collected, four by filtering 2 L of 18.2 MΩ-cm lab-purified water through a Sterivex filter, and four by submitting blank Sterivex filters (opened but unused filters). The blanks were meant to serve as an internal quality control and to base any instances of low biomass (i.e., close to the qPCR detection limit) against. All filters were shipped on dry ice to the Argonne Environmental Sample Preparation and Sequencing Facility at Argonne National Laboratory in Lemont, IL, for analysis, and kept at −80°C until extraction. Notably, we could not guarantee that the same amount of DNA would be collected on each Sterivex filter at each filtered volume (i.e., all 1000 mL samples did not necessarily have the exact same biomass concentrations).

### Laboratory Methods

Standard qPCR and sample preparation methods were used to reduce bias and enable the development of a methodology for produced water sample collection regardless of prior knowledge of biomass concentrations. DNA was extracted from the Sterivex filters using the Qiagen DNeasy PowerWater Sterivex extraction kit (Cat No./ID: 14600-50-NF) following manufacturer instructions. The extracted DNA was used as template for qPCR and Illumina MiSeq sequencing. Each 20 μL qPCR reaction contained 10 μL of SYBR Green Master Mix, 1 μL of Caporaso et al. (2011) 515F forward primer, 1 μL of Caporaso et al. (2011) 806R reverse primer, 7 μL of PCR pure water, and 1 μL of template DNA loaded into each well. The qPCR conditions were as follows: denaturing DNA at 94°C for 3 min followed by a three step cycle 40 times, 94°C for 45 s, 50°C for 60 s, and 72°C for 90 s. All samples were run in triplicate. Positive controls were run in duplicate to ensure a precise standard curve. The qPCR efficiency averaged approximately 96% across the eight point standard curve.

A barcoded primer set adapted for Illumina MiSeq was used to produce PCR amplicon libraries targeting the 16S rRNA encoding gene. After PCR optimization, the V4 region of the 16S rRNA gene (515F-806R) was then amplified using PCR with region-specific universal primers (Caporaso et al., 2011), including sequencer adapter sequences used in the Illumina flowcell and a 12 base barcode sequence that supports sample pooling in each lane (Caporaso et al., 2011, 2012). Each PCR reaction contained 9.5 μL of certified DNA-free MoBio PCR water, 12.5 μL of QuantaBio Accustart II PCR ToughMix (2× concentration, 1× final), 1 μL Golay barcode tagged forward primer (5 μM concentration, 200 pM final), 1 μL reverse primer (5 μM concentration, 200 pM final), and 1 μL of template DNA. PCR conditions were denaturing DNA at 94°C for 3 min, with 35 cycles at 94°C for 45 s, 50°C for 60 s, and 72°C for 90 s, and a final extension of 10 min at 72°C to ensure complete amplification.

Amplicons were then quantified with a plate reader (infinite ^R 200 PRO, Tecan) and PicoGreen (Invitrogen). After quantification, volumes of each product are pooled into a single tube to ensure equimolar amounts of each amplicon. The pool was cleaned using AMPure XP Beads (Beckman Coulter) and quantified using a fluorometer (Qubit, Invitrogen). The molarity of the pool was determined after quantification and diluted down to 2 nM. The pool was denatured and further diluted to a final concentration of 6.75 pM with a 10% PhiX spike for Illumina MiSeq sequencing. Amplicons were sequenced on a 151 base pair × 12 base pair × 151 base pair MiSeq run using customized sequencing primers and procedures.

Resulting Illumina MiSeq data were processed using QIIME2 (Bolyen et al., 2019). Operational Taxonomic Units (OTUs) were mapped at greater than 99% similarity and taxonomy was assigned at the species level. Taxonomic assignments were performed using Silva 132 (Yilmaz et al., 2013) and the dataset was exported to R (R Core Team, 2019) to perform cleaning steps and all statistical analyses. Sequences for each of the 57 samples (including the eight blanks) were scaled to represent percent abundance (i.e., summing all sequences per sample resulted in a value of 100 percent for every sample) so that rarefaction would not occur and limit the dataset by potentially removing operational taxonomic units (OTUs). Sequence reads for each sample were deposited in the National Center for Biotechnology Information (NCBI) Short Read Archive (SRA) under Bioproject PRJNA529810. Data, OTU table, taxonomic table, associated metadata, and code used are available in Shelton and DeVera (2019).

Various methods were used to test the effectiveness of laboratory procedures and data quality for the samples post-16S rRNA gene sequencing, as discussed in the results section. Statistical analyses were performed in R (R Core Team, 2019) with base packages, vegan (Oksanen et al., 2019), ggplot2 (Wickham, 2016), reshape2 (Wickham, 2012), RColorBrewer (Neuwirth and Brewer, 2014), and plyr (Wickham, 2009). Resulting data were analyzed based on Argonne National Laboratory internal qPCR blanks and thresholds and submitted sample set blanks. The results of the Illumina MiSeq sequencing run were used to compare communities of microbes identified in all samples collected to look for differences across the entire sample set and between the smaller volumetric subsets (e.g., the five samples at 1000 mL filtered).

## Results and Discussion

### Using qPCR to Determine if Smaller Volumes of Sample Result in Sequentially Smaller Biomass Concentrations

Fifty-seven samples including 8 external blanks along with one internal laboratory extraction blank were analyzed by qPCR using an eight-point calibration curve (not shown). The results of the qPCR analysis were used to compare relative amounts of biomass in each sample collected and across samples with the same volume of filtrate (e.g., to compare the five 400 mL filtered volume samples). This was performed in order to determine if decreasing sample volume correlated with decreasing biomass concentrations and increasing contamination. Triplicate analyses were performed for each sample, the eight blanks, and one laboratory extraction blank, producing three different Cp values per sample which were averaged (Supplementary Table S1). The Cp or C_*T*_ (threshold cycle) value is the cycle at which the fluorescence achieves a defined threshold and can be useful to understand biomass concentrations in samples. A smaller Cp value is indicative of a larger target expression in a given sample, or more generally, indicative of a larger concentration of targeted DNA per sample. The range of average Cp values for the samples in this study was 25.55 (indicating the largest 16S rRNA copies/μL) for sample JC30 (900 mL) to 40.41 (indicating the sample with the smallest 16S rRNA copies/μL) for sample JC59 (a blank, 0 mL).

Average (not displayed) and median 16S rRNA copies/μL generally displayed a weak trend when compared to filtered volume (Figure 1). The *R*^2^ value for a linear correlation between sample volume and Cp value was 0.0695. Additionally, using a Kruskal-Wallis rank sum test due to a non-normal distribution of Cp value by sample volume, it was confirmed that Cp value is not significantly correlated to volume of sample; there was not a significant relationship between Cp value and sample volume (*p* value > 0.05).

As this is quite an unusual result, it could likely be explained by either (1) variability in biomass concentrations (and also contaminants) in produced water during production from a hydrocarbon well (i.e., biomass concentration varies over short periods of time, such as minutes to hours, during production of water, oil, and gas from a well), (2) the presence of PCR inhibitors disproportionally affecting samples of the same volume, or (3) the volumes filtered are too small to detect differences in microbial density. If biomass concentrations could change across short time scales in hydraulically fractured shale environments, then it is not unreasonable to assume that PCR inhibitor concentrations could change across similar time scales, which could have caused the differences observed in Cp value across identical volumes of sample. PCR inhibitors are chemicals that interfere with the PCR process and are predominantly dissolved or solid organic compounds such as clays, humic acids, phenols, and proteins (Rossen et al., 1992; Abbaszadegan et al., 1993; Ijzerman et al., 1997; Rådström et al., 2004; Schrader et al., 2012). However, previous studies (e.g., Hull et al., 2018; Oetjen et al., 2018) have concluded that the produced water geochemistry of hydraulically fractured shale wells doesn’t change dramatically once in steady state; therefore, PCR inhibitors may also be less variable in concentration once in steady state production. To the authors’ knowledge, there are no studies looking at changes in microbial community composition or geochemistry across short (minutes to hours) time scales in mature hydraulically fractured shale wells, so some variability may be missing in previous studies. Therefore, more investigations should be done to ensure that variability in both water chemistry and biomass does not occur at short time scales in produced water associated with hydraulically fractured shale.

It is also important to note that some of the variability observed in Cp values between samples of the same filtrate volume may be due to the batch of water used during sampling. For instance, carboys 3 and 6 (collected at approximately 0 and 24 h, respectively) only produced samples that had Cp values larger than the suggested detection limit of 30.5. As all of the carboys were identical, it is unlikely that the carboy itself caused these differences. However, carboys one, two and three were all collected minutes apart on day 1 while carboys four, five, and six were collected minutes apart on day 2; this suggests that there may be variation in biomass concentrations in produced waters from shale over very short time scales (e.g., minutes to hours). This will be investigated further in future work.

### Can a Quality Threshold for Low Biomass Samples Be Determined Using Cp Values and Field Blanks?

As simply increasing sample volume was not significantly correlated with increasing biomass concentrations, it would then be ideal to determine a given Cp value that could identify low quality (i.e., low biomass) samples. This Cp would serve as a cutoff threshold where samples with Cp values larger than the threshold are always considered “low-biomass” and potentially could be eliminated from sample sets. In an attempt to determine this Cp value, multiple Cp detection limits were considered when trying to determine if Cp values could define a quality threshold for low biomass samples based on internal laboratory detection limits and externally submitted blank samples.

**FIGURE 2:**
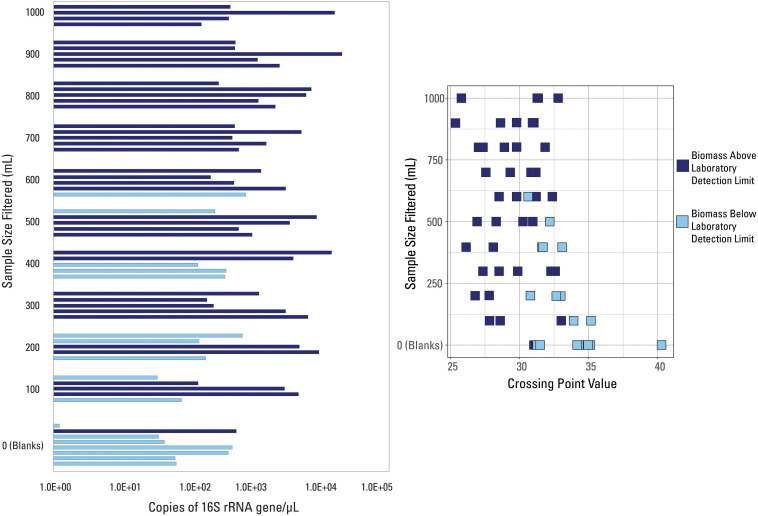
qPCR data visualized with data categorized based on the internal detection limit (library amplification) supplied by the laboratory. Samples are either above (dark blue color) or below (light blue color) the suggested detection limit for library amplification.

The two Cp value thresholds tested were the laboratory’s internal QC threshold, and the Cp value generated based on the blank samples submitted for analysis. Argonne National Laboratory provided information as to which of the 57 samples did not amplify above their internal QC threshold (Figure 2). Samples below the laboratory’s detection limit were generally lower volume samples (200 mL filtered or less), while 7 of the 8 external blank samples also fell below this detection limit (Figure 2). All samples with filtered volumes of 700 mL and greater were above the laboratory detection limit. However, Cp values did not correlate with this detection limit (Figure 2), as many samples had very similar Cp values but were not similarly classified by detection limit (i.e., two samples with the same Cp value were not both below the laboratory detection limit).

Due to the fact that one blank sample was above the laboratory detection limit, this indicates that: (1) this blank sample had a contaminated filter, introducing more biomass than expected; (2) this blank sample was contaminated during the laboratory or analytical processes; and/or (3) this detection limit was not suitable to discern low biomass samples. However, the internal extraction blank submitted by the laboratory had a much larger Cp (i.e., lower value of 16S rRNA copies/μL) than this blank sample, meaning that laboratory, analytical, or background contamination should have been minimal. Therefore, it is likely that this suggested detection limit was inadequate to fully capture poor quality samples and it shouldn’t be used as a threshold.

**FIGURE 3:**
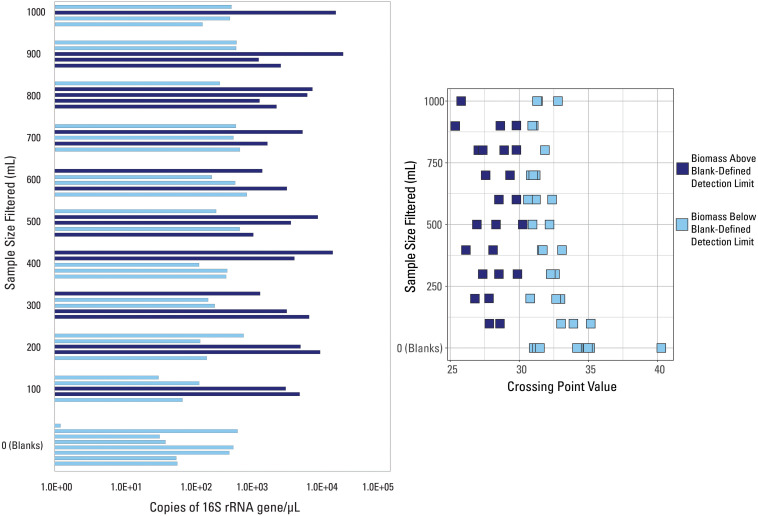
qPCR data visualized with data categorized based on the blank-defined detection limit (Cp = 30.5). Samples are either above (dark blue color) or below (light blue color) the detection limit.

A different detection limit was tested so that every blank sample would fall below it (i.e., all blank samples would be classified as low biomass). The smallest Cp value generated from all eight blanks, Cp = 30.5, was selected as a new detection limit. Samples with an average Cp value greater than that threshold Cp value were deemed below detection limit (*n* = 33) while those with an average Cp value smaller than that threshold value were deemed above the detection limit (*n* = 26), shown in Figure 3. There is no trend in volume filtered when compared to samples falling above or below this new threshold (Figure 3). Every volume sampled had at least one sample above and below the threshold (except the blanks) suggesting variability in the composition of the water sampled, widespread contamination, or that all samples collected were impacted by low biomass.

Glassing et al. (2016) found that their low bacterial biomass samples had Cp values equal to or less than those generated for their no template (i.e., negative) controls. They ranged from 26 to 31 with an average of 29, values that are much smaller than those identified for blanks in this study (Supplementary Table S1). However, many samples in this study had Cp values outside that range; all of the blank samples had an average Cp value greater than 31. This suggests that there may not be one specific Cp value that classifies low biomass conditions or low-quality samples, and that submitting multiple blanks, to establish a well vetted Cp threshold, with a sample set is vital to establishing variation in baseline or non-detect scenarios. Additional work will need to be done to determine if Cp values vary by laboratory, extraction kit, or other circumstances when submitting blanks.

### Comparing the Suggested Cp Threshold to 16S rRNA Illumina MiSeq Sequencing Results

To determine if the suggested blank-defined detection limit (Cp value = 30.5) could filter out poor quality samples, the sequencing data generated for these samples was considered. As discussed previously, as every sample in this study was from the same water source over the course of 24 h, the microbial community composition of every sample (excluding the blanks) should be statistically similar to one another, and the blanks should be statistically dissimilar from the actual samples. The extracted DNA from the 57 samples in this sample set was sequenced so that the microbial community composition of each sample could be compared across the sample set and within its respective volume bin (see Supplementary Table S2 for abbreviated taxonomic table or Shelton and DeVera (2019) for full taxonomic table). Results presented below include the eight blank samples.

**FIGURE 4:**
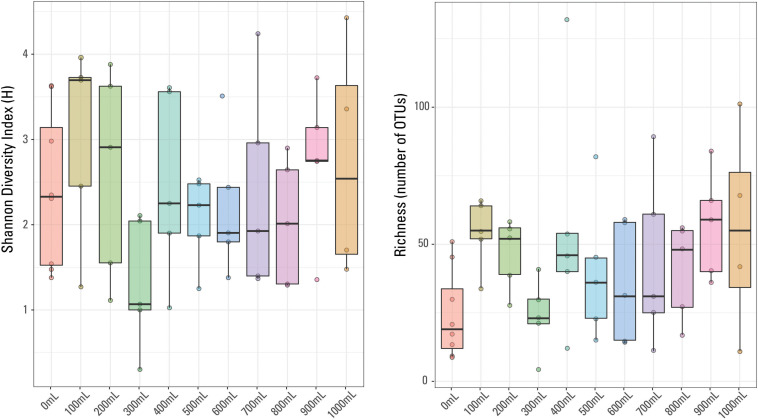
Box plots of Shannon Diversity index and sample richness grouped by volume of sample water filtered.

There were 875 different OTUs identified in the sample set (including the eight blanks), with only one OTU identified in every sample, *Escherichia-Shigella* sp. No other OTUs were identified in every blank or identified in every non-blank sample. The most abundant OTU in each sample did not necessarily dominate (i.e., was present at greater than 20%) the given sample. For example, the most prominent OTU in a given 700 mL sample was present at 4.4% abundance (Acidobacteria, Subgroup 6). Methanogens, thermophilic and halophilic organisms are present, typical of those identified in other waters produced from hydraulically fractured shales (e.g., Kirk et al., 2012; Murali Mohan et al., 2013; Cluff et al., 2014; Wang et al., 2019). Sample richness (or number of OTUs identified per sample) ranged from four OTUs (300 mL sample) to 132 OTUs (400 mL sample). The Shannon Diversity index (H), a measurement of diversity across a sampled microbial community (e.g., Haegeman et al., 2013), ranged from 4.4 (1000 mL sample) to 0.3 (300 mL sample). In general, the 900 mL filtered samples have the highest richness and the 0 mL filtered samples have the lowest sample richness (Figure 4); there is no general trend observed in sample diversity, either variation or similar average values (Figure 4). The median Shannon Diversity index was generally similar for 1000, 800, 700, 600, 500, and 400 mL samples and unexpectedly, the blanks. The largest within-volume variation is observed in 100 and 300 mL samples, possibly suggesting that either these volumes did not capture the representative microbial community of the sampled well and may have been influenced by contaminants or other low biomass artifacts, or the different within-volume samples captured a limited representation of the subsurface microbial community.

**FIGURE 5:**
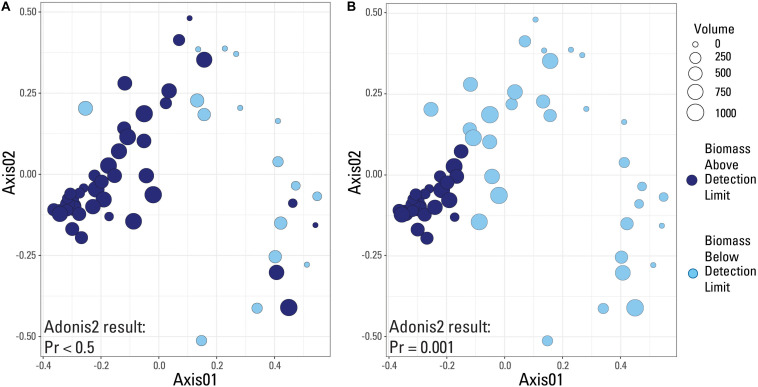
Nonmetric-multidimensional scaling (NMDS) plots using a Bray-Curtis dissimilarity matrix with data categorized based on detection limit used. Samples are either above (dark blue color) or below (light blue color) the given detection limit. **(A)** Laboratory internal detection limit; **(B)** Blank-determined detection limit, Cp value equal to 30.5. Results of an adonis2 test provided on both plots.

A species-based Bray-Curtis distance matrix was visualized using a nonmetric multidimensional scaling plot (NMDS; Figure 5) to determine which samples were most similar to each other (i.e., did samples from the same volume have similar microbial community compositions). An adonis2 test was used to determine if a significant difference in microbial community composition existed between samples above and below the two identified detection limits (laboratory versus submitted blanks). Samples were grouped by either being above or below the library amplification (Figure 5A), or the blank-defined (Cp equal to 30.5; Figure 5B) detection limit. An adonis2 test produced a non-significant *p* value (>0.05) when samples were grouped using the laboratory detection limit, but produced a significant *p* value (0.001) when grouped by the blank-determined detection limit. This suggests that the blank-determined Cp value was able to successfully group samples with significantly different microbial community compositions based solely on biomass concentration. Therefore, samples below this detection limit had a significantly different microbial community composition than samples above this detection limit, meaning that any effects that low biomass samples may have had on the sequencing data may be removed when using this detection limit.

Contamination is the most likely reason that the samples below the detection limit had a different microbial community composition than those above. As contamination more strongly impacts low biomass samples than non-low biomass samples (Salter et al., 2014; Eisenhofer et al., 2019; Karstens et al., 2019; Weyrich et al., 2019), the difference in microbial composition between these two groups of samples may suggest that the detection limit tested successfully separated low biomass samples from the sample set within. However, the microbial ecology of produced fluids of hydraulically fractured wells are known to change over the lifetime of the well (Cluff et al., 2014; Evert et al., 2016), although most of the significant change occurs during the flowback period (typically within the first 2 months). Studies suggest that the microbial ecology becomes stable in mature hydrocarbon-producing wells (e.g., Cluff et al., 2014). Geochemical conditions in established hydrocarbon-producing wells where no injection of outside fluids is occurring also do not vary widely between sampled points, specifically, for the Niobrara Shale (e.g., Hull et al., 2018; Oetjen et al., 2018). The methods used, such as keeping the sample water well mixed and filtering the water across different volumetric batches instead of in succession (i.e., sampling 800, 700, 600, 500 mL in a batch instead of 800, 800, 800, and 800 mL), were done to reduce variability across batches of water collected over the 24 h. Therefore, the microbial ecology should be considerably stable over the sampling time period given the age of the well. Theoretically, similar diversity and richness across samples should also occur if the sampled water was indeed uniform and unchanging over time, as all filtered water originated from the same hydrocarbon well.

When comparing the microbial community composition of samples above and below the threshold defined by the smallest Cp value identified in the blanks, Cp = 30.5, the most obvious difference across the two groups is the abundance of the class *Thermotogae* in the above detection limit samples and the abundance of *Gammaproteobacteria* in the samples below the detection limit (Supplementary Figure S1). OTUs of the class *Thermotogae* are anaerobic, thermophilic, and saccharolytic bacteria that have been associated with thiosulfate reduction to sulfide (Huber et al., 1986), drilling mud in Barnett Shale natural gas wells (Struchtemeyer et al., 2011), hydraulic fracturing flowback water impoundments from the Marcellus Shale (Murali Mohan et al., 2013) and produced waters from oil-producing reservoirs (Salinas et al., 2004; Magot, 2005). *Thermotogae* is not listed in the low-biomass contaminant database defined by Barton et al. (2006); therefore, its presence or absence may be a good indicator to distinguish between high- and low-quality samples, respectively, in samples for this study. The presence of *Escherichia-Shigella* sp. largely explains the dominance of *Gammaproteobacteria* in the below detection limit samples.

### 16S rRNA Sequencing Contaminant Removal and Testing the Proposed Cp Threshold

As a detection limit has been identified, Cp = 30.5, that seemingly was able to distinguish between low biomass samples and non-low biomass samples, the next step was to remove any contamination to see if the above detection limit samples become more similar to each other to test whether or not the blank-defined detection limit was able to successfully capture most of the contamination within the dataset. Therefore, any OTU identified in a blank sample was removed from all other samples in this study.

The blank samples had, on average, 8066 different sequences across 156 different observed OTUs. However, most OTUs had a very low average percent abundance (less than 0.1% abundance) with only one OTU present at greater than 5% abundance, *Escherichia-Shigella* sp., and only 12 OTUs present at greater than 1% abundance: *Nitrososphaeraceae* sp., Acidobacteria Subgroup 6 uncultured bacterium, Acidimicrobiia IMCC26256 uncultured bacterium, *Sporichthyaceae* sp., *Sediminibacterium* sp., uncultured *Flexibacter* sp., *Mucilaginibacter* sp., uncultured rumen bacterium from the class *Kiritimatiellae*, SAR11 Clade Ia sp., *Escherichia-Shigella* sp., uncultured *Chthoniobacteraceae* LD29, and unknown Bacteria sequences. Some of these are organisms commonly identified as contaminants in DNA extraction kits and in the generation of Taq polymerase (e.g., Salter et al., 2014; Chen et al., 2015; Glassing et al., 2016).

Removing all OTUs identified in the blank samples from the rest of the sample set reduced the total number of OTUs in the remaining 49 samples to 719. The minimum and maximum number of sequences per sample changed from 447 and 89,394, respectively, to 28 and 40,948, respectively. The impact of removing the contaminant OTUs identified in the blank samples can be observed in Table 1.

Samples in Table 1 are organized as either above or below the detection limit of Cp = 30.5 (as discussed in previous sections). Fewer contaminants were present in the samples classified above the Cp detection limit than those below the detection limit, supporting the use of the smallest Cp value generated for the blanks as a good threshold for determining data quality. Theoretically, contamination could affect low biomass and non-low biomass samples in the same way but impacts of that contamination would be greater in low biomass samples, as there is less real sample DNA (e.g., Salter et al., 2014) or the contaminants are a much larger proportion of the sample when below the detection limit. Therefore, one would expect that the number of OTUs removed per sample and/or the relative number of sequences per sample removed due to contamination should be much higher in low biomass samples than in non-low biomass samples.

A study by Karstens et al. (2019) used serial dilutions of a mock community to investigate how biomass concentration and contamination are related. Their experiment found that contamination increased with decreasing starting biomass concentration, or that increasing dilution increased contamination. This is not in agreement to what we observe here; we see no relationship with smaller volumes of water filtered and contamination. Increasing sample volume, which should theoretically be tied to increasing biomass volume, is not related to decreasing contamination or higher-quality samples. However, the samples with Cp values greater than 30.5 do have a greater percentage of their OTUs comprised of contaminant OTUs than those above this detection limit, but the inverse is generally true for contaminant sequences per sample. It appears that natural systems are harder to decipher than mock community dilutions like those presented in Karstens et al. (2019), and that it is not appropriate to assume that collecting a greater volume of sample will result in greater amounts of biomass and thus, fewer impacts from contamination.

**FIGURE 6:**
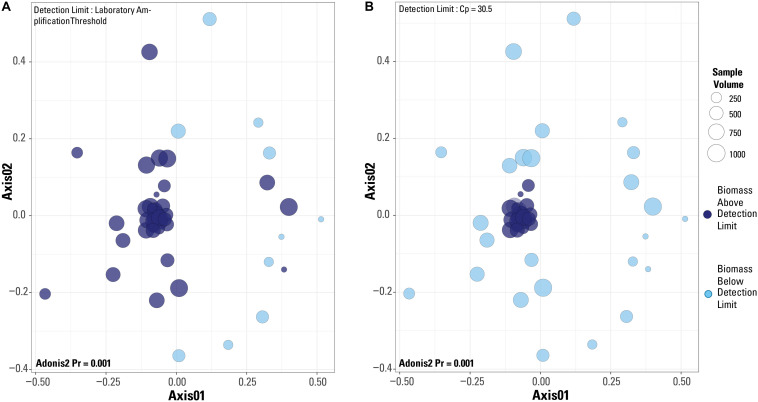
Non-metric multidimensional scaling plots of a Bray-Curtis distance matrix based on the dataset after blanks and contaminant removal and normalization of sequences per sample. Plots are of the two different detection limits described in the test. **(A)** Detection limit is based on the laboratory’s internal amplification threshold. **(B)** Detection limit is based on blank-defined detection limit, Cp = 30.5. Results from an adonis2 test are provided on both plots.

When a Bray-Curtis distance matrix of the blank-removed dataset was visualized via an NMDS, significant clustering is observed (Figure 6). The two different detection limit scenarios are illustrated, with the laboratory’s library amplification threshold plotted in Figure 6A and the blank-defined threshold (Cp = 30.5) used as the detection limit in Figure 6B. An adonis2 test produced a significant *p* value (*p* = 0.001) for both detection limit even though visually, it appears that the blank-defined threshold more successfully captures the samples with extreme microbial community composition similarity (as many samples plot on top of each other). The samples that cluster near the origin of the plot all have very similar microbial community compositions, which is expected for samples representing the composition of the single well sampled for this study. In Figure 6A, there is no clear clustering of samples based on detection limit even though the two groups cluster significantly according to an adonis2 test. Although the two groups in Figure 6A (above and below the detection limit) are significantly different, the similarity of the samples above the detection limit is greater in Figure 6B, or when Cp = 30.5 is used as the detection limit.

### The Role of Blanks in Low Biomass Samples

As the blank-defined detection limit proved to be better at differentiating low biomass samples from a sample set, it is clear that the submission of external blanks is critical when sampling a potentially low biomass environment. Additionally, the development of this detection limit was highly dependent on the number of blanks submitted with a sample set. Removing any of these eight blanks could change the Cp value used as the detection limit for these scenarios and could therefore easily change which samples are classified as above or below a suggested detection limit. It is therefore critical to submit a large number of blanks when sampling; future work will focus on how many blanks proves most successful in creating a usable Cp detection limit.

Furthermore, even after contaminant removal, there was still variability in microbial community composition across the remaining 49 samples. This suggests that simply removing contaminants from low biomass samples may not improve the samples enough to be worthwhile to include in data analysis. We suggest that it is imperative to differentiate from samples impacted by their low biomass signature and remove them from sample analysis.

Overall, these results suggest that increasing sample volume does not necessarily directly relate to increasing biomass concentrations (possibly due to increased presence of PCR inhibitors) or the likelihood for a sample to be close to or at the detection limit for qPCR. These results also imply that no relationship between sample volume and microbial community composition exist in this sample set. The samples most affected by the removal of contaminant OTUs generally fell below the detection limit, suggesting that using the smallest Cp value in all submitted blanks as a detection limit may serve as a good metric for weeding out low biomass samples that may result in misleading or incorrect data. However, this is highly dependent on the number of blanks submitted. Even though all samples collected were from the same produced water source, the microbial community compositions before and after contaminant removal varied widely across the sample set. This suggested that simply implementing contaminant removal techniques for samples may not be enough to prove worthwhile in downstream analysis, as many samples in the sample set with the largest DNA concentrations had significantly similar microbial community compositions.

### Microorganisms Identified in High Biomass Samples

There were 24 samples that had adequate biomass concentrations (i.e., fell above the suggested Cp threshold of 30.5). Although investigating the identified microbial community composition of these 24 samples was not the focus of this study, expanding on the communities identified would be useful to further characterize the microbes present in the Niobrara Shale. After contaminant removal, the major taxa identified in these 24 samples were *Thermovirga* spp. (10.2% average abundance across the 24 samples), uncultured *Methanothermobacter* (9.2% average abundance), *Caldanaerobacter* spp. (8.3% average abundance), and *Thermoanaerobacter* spp. (5.7% average abundance). Many of the identified OTUs are thermophilic, methanogens, or halophilic organisms.

The orders *Methanobacter* (uncultured *Methanothermobacter*) and *Thermoanaerobacterales (Thermoanaerobacter* spp.), and the classes *Clostridia* (*Caldanaerobacter* spp. and *Thermoanaerobacter* spp.) and *Synergistia* (*Thermovirga* spp.) have been previously identified in early (i.e., in production for fewer than 100 days) Niobrara produced waters (Hull et al., 2018; Oetjen et al., 2018; Wang et al., 2019). *Thermoanaerobacterales* are sulfidogenic organisms, potentially indicating the presence of sulfate in these produced fluids and the potential for well souring (Davis et al., 2012). *Methanothermobacter* is typically associated with hydrogenotrophic methanogenesis (e.g., Wang et al., 2019), and its presence in these samples could indicate the potential for ongoing methane production in the Niobrara Shale that could be stimulated.

Similar to Hull et al. (2018) and Oetjen et al. (2018), *Halanaerobium* was not detected in any samples within, perhaps providing further evidence that the microbiology of hydraulically fractured shales are not uniform and may be specific to other formation conditions, such as salinity (Kondash et al., 2017). Hull et al. (2018) also identified an abundance of *Methanothermobacter* in Niobrara Shale horizontal produced fluids at a different location in the DJ Basin, indicating that wide-spread enhancement of methanogenesis across the shale may be possible, and that hydrogenotrophic methanogenesis may be the major metabolic pathway for methane generation. Further work on the genomics of Niobrara Shale produced waters would be necessary to confirm this hypothesis.

The four predominate orders identified in this study were not identified in any of the samples below the blank-defined detection limit. This indicates two things: (i) that these four classes could be used as indicator species for waters produced from late-time series wells in the Niobrara Shale, and (ii) that, again, the established blank-determined threshold was valid for use in this study. The organisms identified in abundance in the samples above the low biomass threshold are typically identified in extremely similar environments (i.e., the Niobrara Shale). Our analysis indicates these microorganisms are present in mature steady-state formation water long after the flowback period. Although the organisms identified in the low biomass samples can be loosely tied to oil and gas production, the four communities discussed above were not abundant in those samples. Therefore, this provides further evidence that low biomass samples must be screened and that this study may represent an adequate sampling plan to capture organisms present in low biomass environments. The microbial ecology of formation water associated with the Niobrara Shale will be expanded on in future research.

### Sampling Plan Recommendations

Theoretically, increasing the volume of water sampled should increase the volume of biomass collected, but this relationship was not observed in this study. Overall, these results suggest that increasing sample volume does not necessarily directly relate to increasing biomass concentrations or the likelihood for a sample to be close to or at the detection limit for qPCR. No relationship between sample volume and microbial community composition exist in this sample set. The samples most affected by the removal of contaminant OTUs generally fell below the detection limit, suggesting that using the smallest Cp value in all submitted blanks as a detection limit may serve as a good metric for filtering out low biomass samples, where their inclusion may result in misleading or incorrect data. However, this is highly dependent on the number of blanks submitted. Even though all samples collected were from the same produced water source, the microbial community compositions before and after contaminant removal varied widely across the sample set. This suggested that simply implementing contaminant removal techniques for samples may not be enough to prove worthwhile in downstream analysis, as many samples in the sample set with the largest DNA concentrations had significantly similar microbial community compositions.

This suggests that researchers may be able to collect many lower volume samples (e.g., 500 mL compared to 1000 mL) and get the same quality data (i.e., a representative sample), which could save time in the field. One could argue that collecting a larger volume of sample over a longer time is needed to fully capture a truly representative sample of the produced water microbial community, but the results within do not support that a large sample is necessary. If there is indeed variability in the microbiology of produced fluids from shale wells over short time periods like those sampled in this study (hours to days), collecting many smaller volume samples would capture this variability better than a few large volume samples, because the lower volume samples also represent smaller points in time. Additionally, this could also suggest that it is more prudent to obtain multiple samples in collected and composite time-integrated water samples.

When collecting samples of unknown biomass concentrations, we recommend simply collecting multiple lower volume samples over a few large volume samples. This is because potential variability in biomass across short time scales in a production well may be more adequately captured in smaller volume samples. We suggest submitting multiple blanks and using the smallest Cp value as a cutoff for usable data in downstream analyses. Simply submitting one blank or only using an extraction blank may not be adequate to account for any contamination or underlying variation in sequencing results due to low biomass conditions. Setting a conservative detection limit using a large number of internal and external blanks is the key to obtaining reliable data. If all Cp values are below the defined threshold suggested by blank submission, other methods such as sample pooling (i.e., taking multiple samples from a sample site and pooling the extracted DNA from those samples into one sample) may be required to overcome the limitations of low biomass settings. Additional research on the reproducibility of multiple low volume samples compared to a few large volume samples in environments outside produced water is necessary. Testing this hypothesis on shotgun metagenomic data would also be useful for future studies as well as investigating changes in microbial community composition over short time periods (hours to days) in mature oil and gas wells.

## Data Availability Statement

Sequence reads for each sample were deposited in the National Center for Biotechnology Information (NCBI) Sequence Read Archive (SRA) under BioProject PRJNA529810.

## Author Contributions

JS developed the research question. JS, LR, and AJ devised the research plan. JS, MB, and CD performed field work. JS and EB performed data analysis and interpretations. All authors drafted the manuscript.

## Conflict of Interest

The authors declare that the research was conducted in the absence of any commercial or financial relationships that could be construed as a potential conflict of interest.

## Supplementary Material

TABLE S1 Taxa identified in the raw (blanks included) dataset. Taxa are presented to the class level only.
