# Supplementary figures and images for: Repetitive Sampling and Control Threshold Improve 16S rRNA Gene Sequencing Results From Produced Waters Associated With Hydraulically Fractured Shale
Source: Front Microbiol. 2020 Sep 11;11:536978. doi: 10.3389/fmicb.2020.536978 (PMC7518088; doi:10.3389/fmicb.2020.536978)

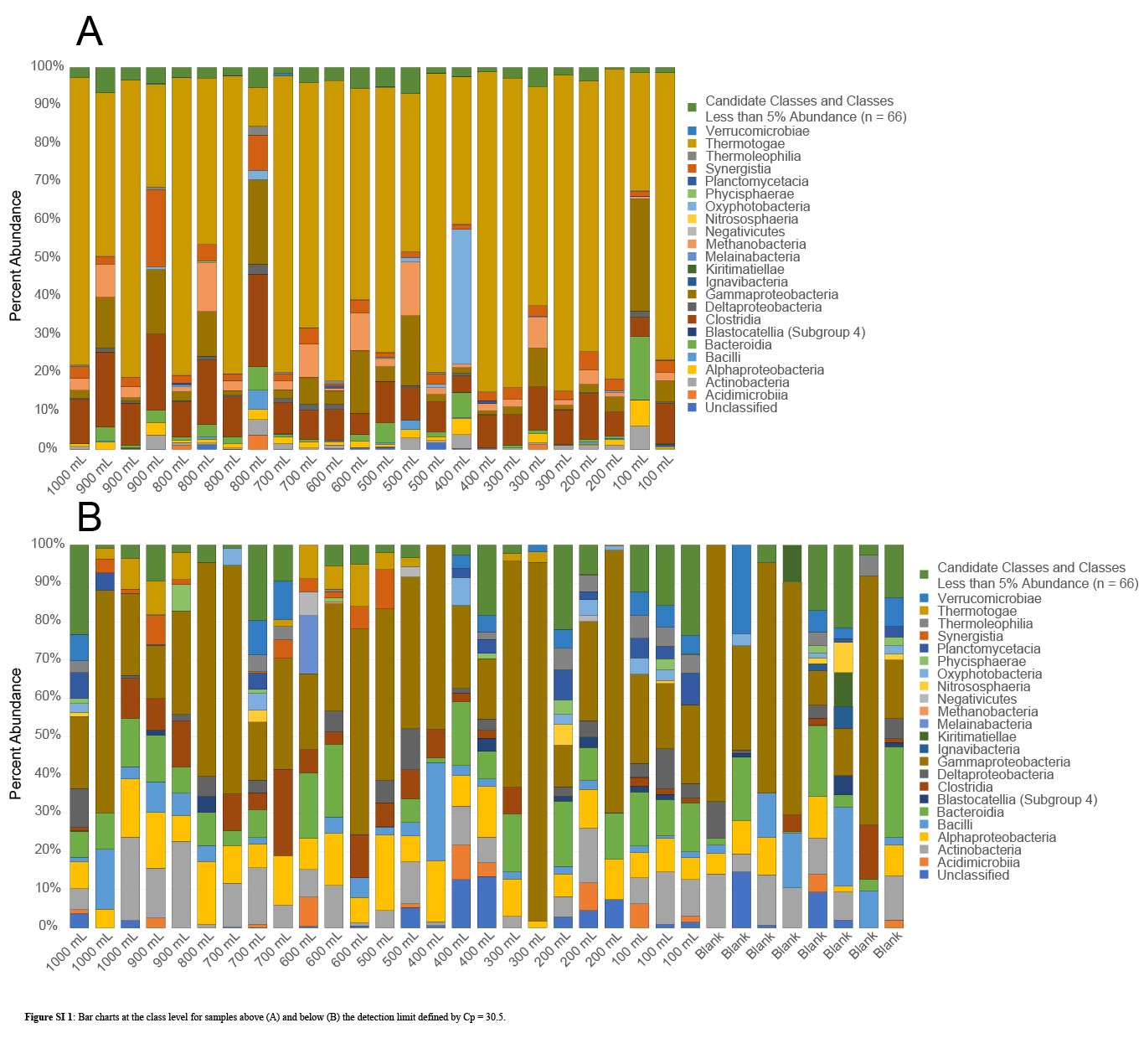

Supplement: Supplementary file 2 [file Image_1.JPEG]
